# Editorial: Unraveling the links between nutrients and metabolic dysfunction-associated liver disease: insights and implications

**DOI:** 10.3389/fnut.2024.1525180

**Published:** 2024-11-27

**Authors:** Bruno Ramos-Molina, Claudia Tovar-Palacio, Ivan Torre-Villalvazo, Md. Wasim Khan

**Affiliations:** ^1^Obesity, Diabetes and Metabolism Research Group, Biomedical Research Institute of Murcia (IMIB), Murcia, Spain; ^2^Dirección de Nutrición, Instituto Nacional de Ciencias Médicas y Nutrición, Mexico City, Mexico; ^3^Departamento de Fisiología de la Nutrición, Instituto Nacional de Ciencias Médicas y Nutrición, Mexico City, Mexico; ^4^Department of Medicine, University of Illinois at Chicago, Chicago, IL, United States

**Keywords:** MASLD, metabolic dysfunction associated steatotic liver disease, MASH and liver fibrosis, nutrition, lifestyle, biochemistry and metabolism

Metabolic dysfunction-associated steatotic liver disease (MASLD) and metabolic dysfunction-associated steatohepatitis (MASH) are the most prevalent liver diseases worldwide. Fortunately, in recent years, our understanding of the physiopathology of both diseases has expanded significantly, particularly regarding the roles of diet, lifestyle, and metabolic regulation. This Research Topic of studies provides critical insights into these aspects, presenting multifaceted approaches encompassing dietary and lifestyle influences, metabolic connections, therapeutic agents, and diagnostic advances. These studies underscore the potential of integrated strategies in preventing and managing liver disease.

A key theme within this collection is the impact of dietary intake and specific nutrients on liver health. Wang et al. conducted a meta-analysis on nearly half a million participants, revealing a protective effect of fruit and vegetable consumption against MASLD. They showed that higher intake of fruits and vegetables correlated with a reduced risk of MASLD, underscoring the importance of antioxidant-rich foods for liver health. The strength of this association varied by demographic factors, highlighting the necessity for population-specific dietary guidelines. The antioxidant theme continues in studies examining specific dietary components. Complementing these findings, Majeed et al. performed a clinical trial to evaluate the effects of a supplement containing garcinol, curcuminoids, and piperine in patients with mild to moderate MASH. This randomized, double-anonymized, placebo-controlled study suggests that this herbal combination may offer therapeutic benefits by targeting inflammation and oxidative stress. This supplementation approach could be a beneficial adjunct to conventional treatments, especially for patients in the early stages of liver disease. Using a different approach, Ravaut et al. examined the impact of the ketogenic diet on reversing steatosis. This study explains how low-carbohydrate, high-fat diets might influence liver status in diet-induced MALSD mice. Initial analysis in male mice indicates that the ketogenic diet could support the reduction of hepatic steatosis, suggesting that carbohydrate restriction might help address metabolic liver disease. While promising, further research is needed to understand better the ketogenic diet's effects over longer durations and its potential for translation to human applications.

In a systematic review, Zhang X. et al. also investigated the Dietary Inflammatory Index (DII) and its association with MASLD. Analyzing data from two million participants, they found that higher DII scores, indicative of pro-inflammatory diets, are linked to a significantly elevated risk of MASLD. These findings highlight the importance of anti-inflammatory diets, emphasizing the role of dietary choices in reducing inflammation and, subsequently, MASLD risk. Complementing these findings, another study evaluated the oxidative balance score as a predictive measure for MASLD. This research emphasizes that dietary and lifestyle modifications aimed at improving oxidative balance may serve as valuable preventative measures for MASLD, further reinforcing the role of antioxidant-rich foods in liver health.

In studies addressing dietary biochemistry, Cai et al. explored the association between low serum folate levels and advanced liver disease stages in MASLD. They reveal that reduced levels of folate and 5-MTHF correlate with increased liver steatosis and fibrosis, pointing to the potential for folate supplementation as a modifiable factor in MASLD management. In addition, Xiang et al. investigated the relationship between blood chromium levels and hepatic steatosis, assessed via liver ultrasound transient elastography, using data from the National Health and Nutrition Examination Survey (2017-2020). The authors found a significant inverse association between blood chromium concentrations and the presence of hepatic steatosis, suggesting that higher chromium levels may be linked to a reduced risk of fatty liver. These findings support the potential of chromium as a modifiable factor in managing liver health, though further studies are needed to confirm causality.

In the area of therapeutic interventions to combat MASLD progression, Zhou et al. presented innovative research on peach gum polysaccharides (PGPs), investigating their antioxidant and hepatoprotective properties in models of alcohol-induced liver injury. Their findings suggest that PGPs can alleviate oxidative stress and inflammation while modulating amino acid metabolism, positioning PGPs as promising, plant-based candidates for treating liver disease with minimal toxicity. Further complementing these therapeutic insights is Ruan et al.'s review on metformin, a well-known diabetes medication that may also have promising applications for MASLD and hepatocellular carcinoma (HCC). The review highlights metformin's beneficial effects on lipid and glucose metabolism, AMPK activation, and gut microbiota modulation. Its anti-inflammatory properties suggest metformin could play a multifaceted role in managing liver disease, particularly in fibrosis prevention and HCC treatment. This review proposes that metformin, traditionally used in diabetes management, may have a broader impact on chronic liver disease due to its regulatory effects on metabolic and inflammatory pathways.

On the topic of lifestyle-based therapeutic strategies, Mambrini et al. advocated for a multidisciplinary approach in treating MASLD, focusing on personalized dietary and exercise interventions. Their review emphasizes the importance of improving insulin sensitivity and metabolic flexibility through customized lifestyle changes, highlighting the need for collaboration among dietitians, exercise specialists, and physicians. This comprehensive approach underscores the potential for tailored lifestyle modifications to impact patient outcomes and positively reduce disease progression in MASLD. Charlot et al. contributed further to our understanding of dietary approaches with their comparative study on hypercaloric low-carbohydrate, high-fat diets (LCHFD) vs. Western diets in mice. Their findings suggest that LCHFD may be protective against MASLD, despite equal caloric intake, indicating that carbohydrate reduction, rather than simple calorie restriction, could be a key factor in managing obesity-linked liver disease. This study encourages a re-evaluation of dietary guidelines, advocating for low-carbohydrate dietary patterns as an effective strategy for liver health.

Advances in diagnostic tools and nutritional assessment are also explored in this collection. He et al. examined malnutrition in patients with liver cirrhosis, comparing different assessment tools to address nutritional deficits more effectively. Their study suggests that the RFH-NPT screening tool is the most practical for malnutrition screening. At the same time, the GLIM criteria prove to be highly accurate for diagnosing malnutrition in cirrhotic patients. By highlighting the importance of early detection and intervention, this research underscores the need for improved nutritional assessment to enhance patient outcomes in advanced liver disease. Chen et al.'s mini-review synthesizes current knowledge on MASLD diagnosis and lifestyle interventions. They discuss the diagnostic value of combining serologic and imaging markers and emphasize the preventive role of diet and physical activity in MASLD management. This review calls for further research into MASLD pathogenesis and underscores the importance of individualized approaches, echoing the necessity of integrating diagnostic precision with personalized lifestyle interventions for optimal liver disease management.

Finally, in a systematic review, Zhang D. et al. explored the bidirectional relationship between *Helicobacter pylori* (*H. pylori*) infection and MASLD, underscoring their complex interplay with metabolic syndrome. Through a meta-analysis of 34 studies, the authors reveal that *H. pylori* infection increases the risk of MASLD and vice versa, establishing that these two conditions exacerbate each other. By linking *H. pylori* to metabolic factors that underlie MASLD and metabolic syndrome, this study opens new avenues for understanding the pathogenic overlap and invites further investigation into mechanisms by which bacterial infection might influence liver health.

In summary, this Research Topic brings together pioneering studies that reinforce the critical role of dietary, lifestyle, and therapeutic interventions in managing MASLD and related liver conditions. From antioxidant-rich foods and dietary patterns to personalized treatment approaches, these articles present a robust framework for understanding the multifaceted pathways through which liver health can be supported. By integrating evidence from nutrition, pharmacology, and lifestyle medicine, this Research Topic advances a holistic perspective, inviting healthcare professionals and researchers to consider comprehensive strategies that encompass prevention and treatment in the fight against liver disease.

